# Supplementary material for: The evolution of life-history theory: a bibliometric analysis of an interdisciplinary research area
Source: Proc Biol Sci. 2019 Mar 27;286(1899):20190040. doi: 10.1098/rspb.2019.0040 (PMC6452081; doi:10.1098/rspb.2019.0040)
Supplement: Supporting Information [file rspb20190040supp1.pdf]

Supporting Information for Nettle and Frankenhuis, 'The evolution of life history theory'

Contents

Section 1. 'Life history theory' and alternative search terms 2

Section 2. Mapping the whole of the literature 4

Section 3. Finer-grained division of time 5

Section 4. Co-citation maps 6

Section 5. Varying the cluster resolution parameter 8

Section 6. Lists of sampled papers by cluster 9

Section 7. Information on the clusters (tables S1 and S2) 14

References cited (this document) 16

Section 1. 'Life history theory' and alternative search terms

We based our study on the topic search term 'life history theory' because we were interested in the emergence and use of that particular label. However, it is clear that this search term does not capture all of the literature on life history evolution. Indeed, many works commonly thought of as establishing canonical 'life history theory' ideas did not in fact use that term and so are not captured by the search (e.g. [1,2]). Stearns [3] estimated that there had been 52 papers on life history evolution by 1980, whereas our search captured only one [4]. Our strategy therefore under-samples the early theoretical work on which life history theory is based. Our study is therefore best thought of as a study of the label 'life history theory', rather than a review of the whole literature that has contributed to our understanding of the evolution of life history traits.

We did, however, also explore a broader search strategy designed to capture more of the literature on life history evolution. We searched Web of Science for '("life history theory" OR "life history evolution" OR "life history strategies")'. This search was performed on February 21st 2019, several months later than the searches reported in the main paper. This search returned 7185 documents, almost four times as many as the 'life history theory' search. We also constructed maps of this broader literature, again dividing into two time periods, up to and including 2010, and post-2010. These maps are shown in figure S1.

The broader search appears to return a much greater range of research on plants, invertebrate animals, reptiles, amphibians and fishes than the 'life history search'. Moreover, research on humans constitutes a smaller proportion of the data. This suggests that the label 'life history theory', as opposed to 'life history evolution' or 'life history strategies', has become particularly widespread in research on humans, whereas related ideas appear in other parts of biology under slightly different labels.

The map up until 2010 is basically radial in structure, though its 'arms' are longer than in the 'life history theory' search, whilst the post-2010 map is more linear in structure. This shift over time echoes figure 2 of the main paper. Once again we see human research concentrated at one end of the continuum and non-human work at the other, though the human research constitutes a smaller proportion of the total on this broader search than for 'life history theory'. There are seven clusters in both time periods for this broader search. Establishing what these contain would be a full study in its own right. Some of them are taxonomically based: in both time periods there appears to be a cluster of work on ocean fish and fisheries management (dark blue); and one on freshwater fish ecology and evolution (light blue). Our impression is that that in this broader search, more of the clusters are genuinely taxonomically mixed. For example, the research in the purple clusters in both maps spans multiple taxa. However, in both time periods the research on humans is concentrated into a single cluster, shown in green. The three distinct clusters of human research of figure 2B in the main paper do not separate here, though the relative positions of different styles of human research does correspond to that figure (i.e. more 'psychological' research is generally positioned further away from the non-human clusters).

A

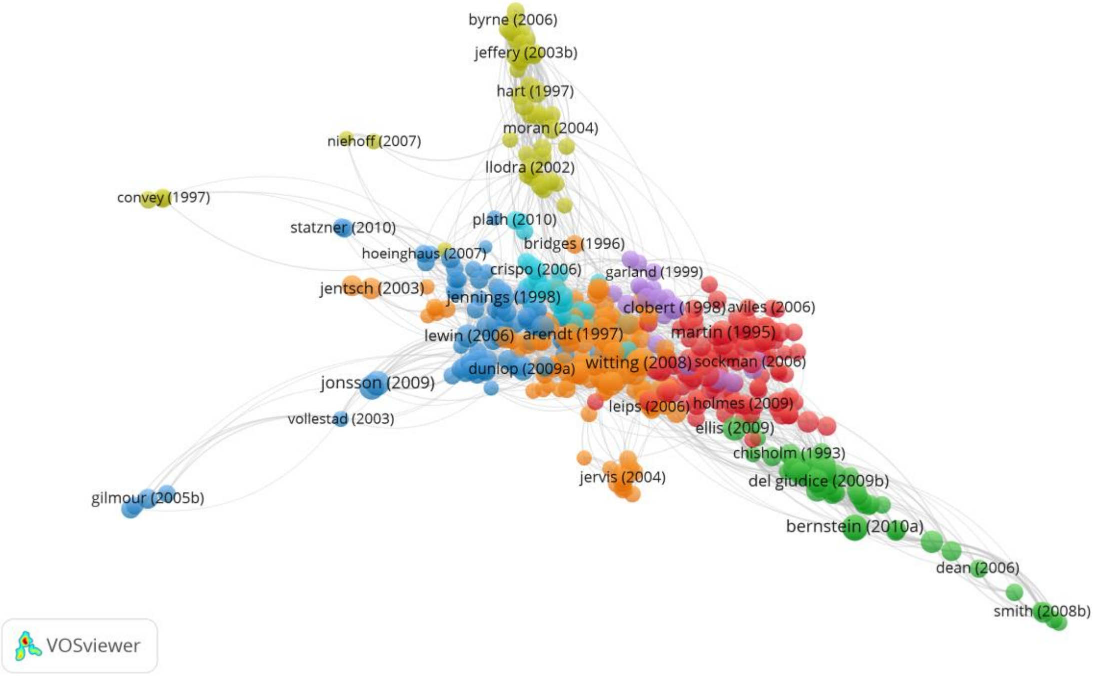

B

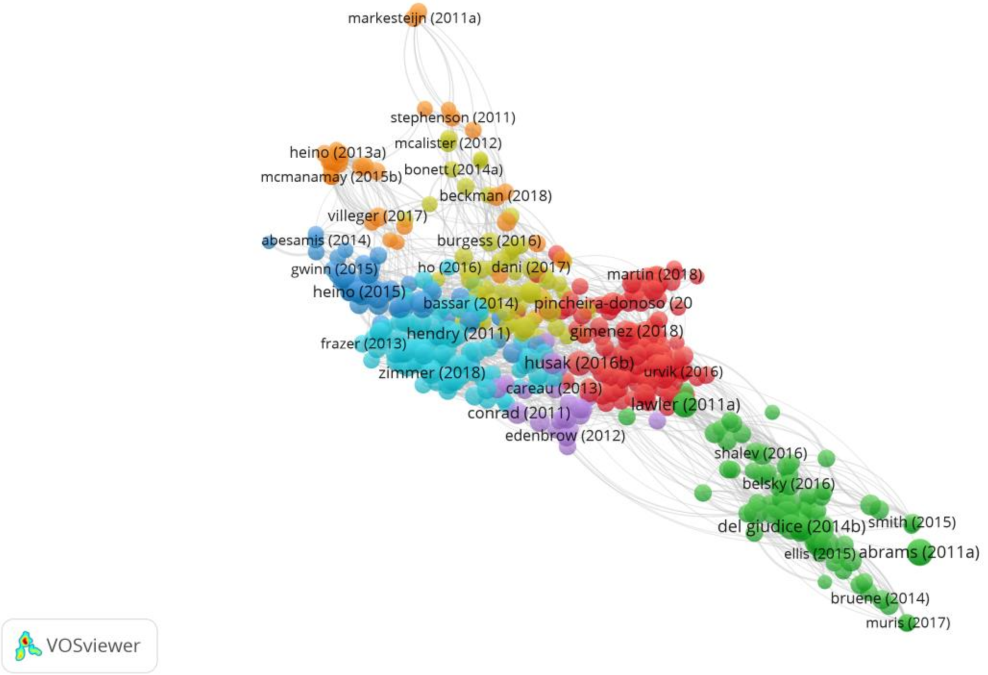

Figure S1. Bibliographic coupling maps of the broader literature search '"life history theory" OR "life history evolution" OR "life history strategies", using the same parameter values as the maps in figure 2 of the main paper. A. Literature up to and including 2010. B. Literature post-2010.

Although the results of the broader search are different in detail from those of the 'life history theory' search, they do support the notion of an increasingly linear literature, with 'life history' research on humans sequestered at one end and not strongly connected to corresponding work on non-human organisms.

We also searched Web of Science for 'pace of life' (see Discussion of main paper). This returned 407 hits, all only 22 were also in the 'life history theory' set. The papers in the 'pace of life' set tend to draw on the 'fast-slow continuum' and related ideas. For example, 123 of the 407 mention 'fast' in their title or abstract, and 113 mention 'slow'. As we argue in the main paper Discussion, the research programme of clusters B2 and B5 more logically belongs with the non-human 'pace of life' literature than it does with the literature on life history theory.

Section 2. Mapping the whole of the literature

For our analysis in the main paper, we divided the literature into two time periods, up to and including 2010, and post-2010. If instead we use the whole of the 'life history theory' literature for mapping, we obtain a map with a similar general shape to the post-2010 map in the main paper (figure S2). As in the main paper, the human research is concentrated along one end of the line, with the 'dark triad' research most distant from the rest of the literature. This analysis produces four clusters using the same cluster resolution as in the main paper. These clusters appear to predominantly correspond to: birds and mammals (red); fish, insects and other taxa (blue); human evolution (dark green); and human developmental/personality psychology (mid green). The human developmental/personality psychology cluster amalgamates clusters B2 and B5 of the post-2010 map from the main paper: spatially, these two research areas still separate, but they are not assigned separate clusters in this overall analysis.

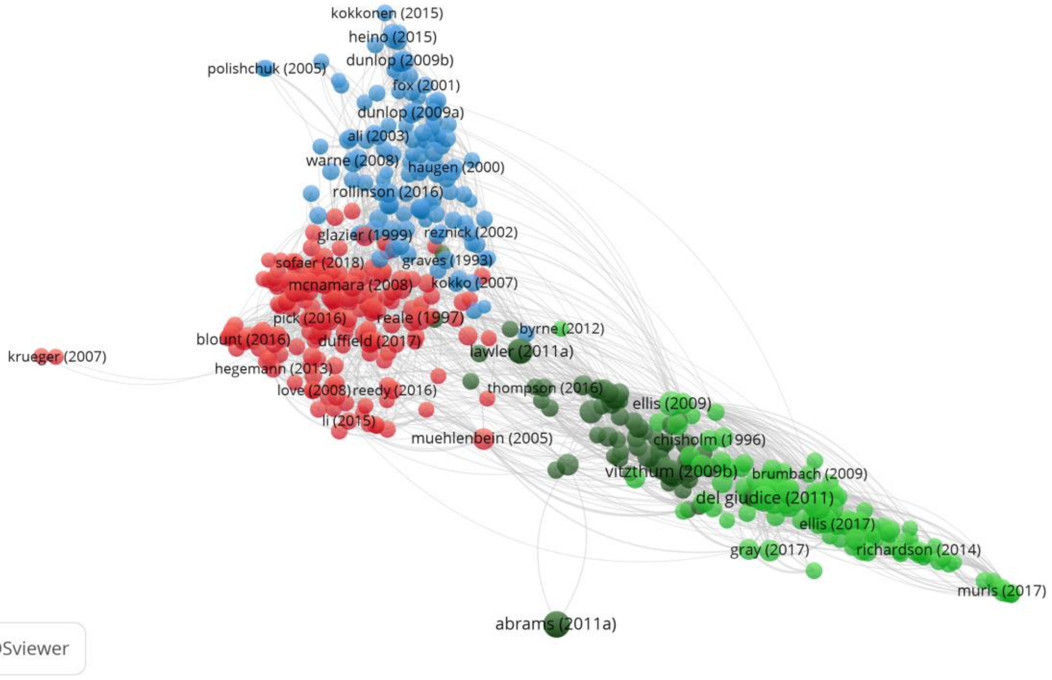

Figure S2. Bibliographic coupling map including both the earlier (up to and including 2010) and recent (post-2010) literature together. Parameter settings are as for figure 2 of the main paper.

Section 3. Finer-grained division of time

As well as the binary division of time used in the main paper, we also divided the 'life history theory' data into four time categories containing roughly equal numbers of records: up to and including 2004 (n = 458); 2005-2010 (n = 453); 2011-2014 (n = 423); and 2015-2018 (n = 508). Figure S3 shows bibliographic coupling maps of each of these four time bins using the same parameter values as the maps in the main paper.

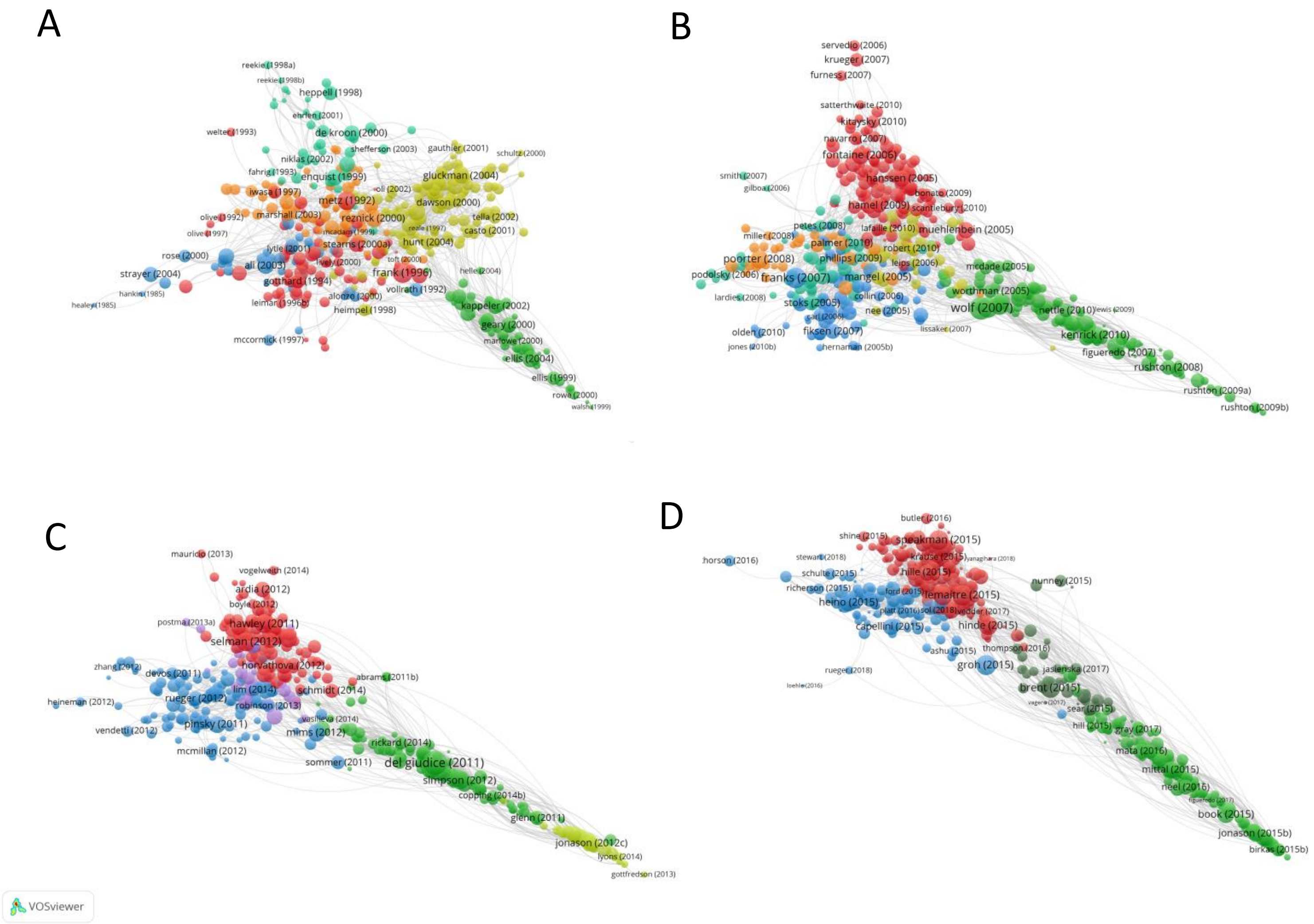

Figure S3. Bibliographic coupling maps for the time periods up to and including 2004 (A); 2005-2010 (B); 2011-2014 (C); 2015-2018 (D). Parameter values are as for figure 2 of the main paper.

The sequence of maps in figure S3 clearly shows the increasingly linearity of the literature over time. It also shows the increasing variegation of the human research: in the first and second time periods, there is only one cluster of human research. In the third time period, the 'dark triad' cluster separates from the human development cluster, although the two are combined again in the fourth time period. The division between the human evolution and human developmental/personality psychology is only detected by the clustering algorithm in the most recent time period. However, although not yet marked enough to be detected by the clustering algorithm, the difference between the three kinds of human life history theory research on humans existed from the very earliest time period. For example, in the period prior to 1995, papers by Helle et al. [7], Chisholm [8] and Rushton [9] respectively already exemplified the styles of research that would later become the three human clusters. The relative positions of these three types of research on the map, and their relative distance from non-human research, are completely consistent across the time periods.

Section 4. Co-citation maps

An alternative to the bibliographic coupling method we use in the main paper is co-citation analysis. Here, the nodes on the map are the papers cited by the papers found in the literature search. The links are formed by being cited by the same source. Co-citation analysis should produce similar representations of the structure of the field as bibliographic coupling [10]. We repeated our mapping exercise from the main paper using co-citation analysis instead of bibliographic coupling. We used a minimum number of citations of eight for a node to be included. We also set the cluster resolution parameter to 1.0 instead of 0.8 as for the bibliographic coupling: the number of clusters found at any given resolution was fewer in the co-citation analysis. Other parameter values were as for the bibliographic coupling. The resulting maps were as shown in figure S4.

The same basic generalizations hold as for the bibliographic coupling maps, especially the increasing linearity of structure of the map. In the earlier time period, five clusters were detected; as in the main paper, all the human research was in one of these. In the later time period, the clustering identified six rather than five clusters. Three of these were identical to the bibliographic coupling clusters (the three human clusters, B2, B4 and B5). The non-human literature was divided into three clusters here rather than the two in the bibliographic coupling map.

A

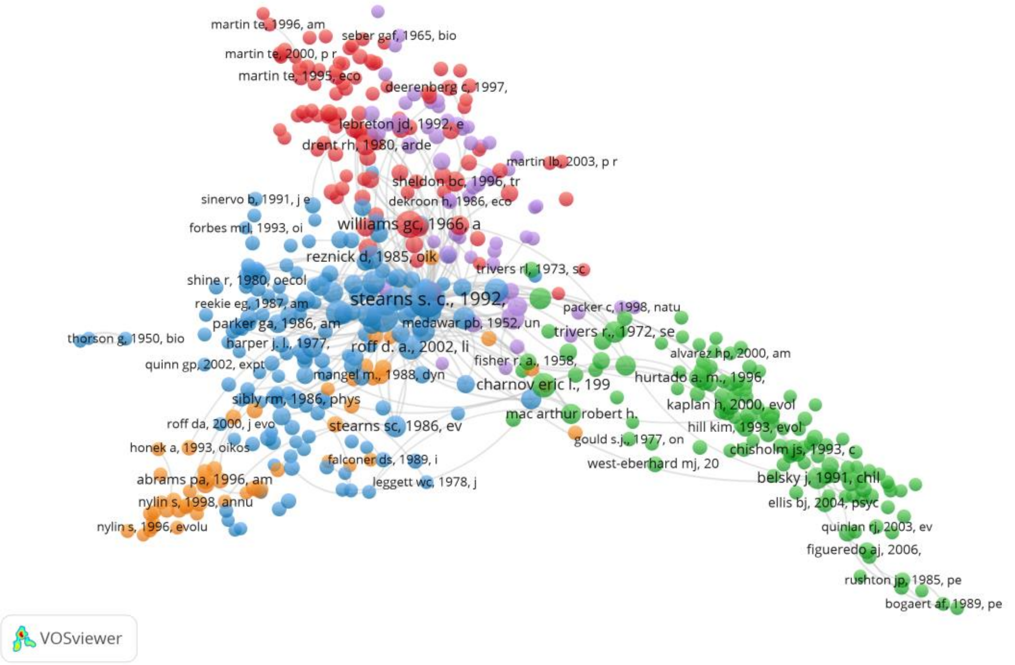

B

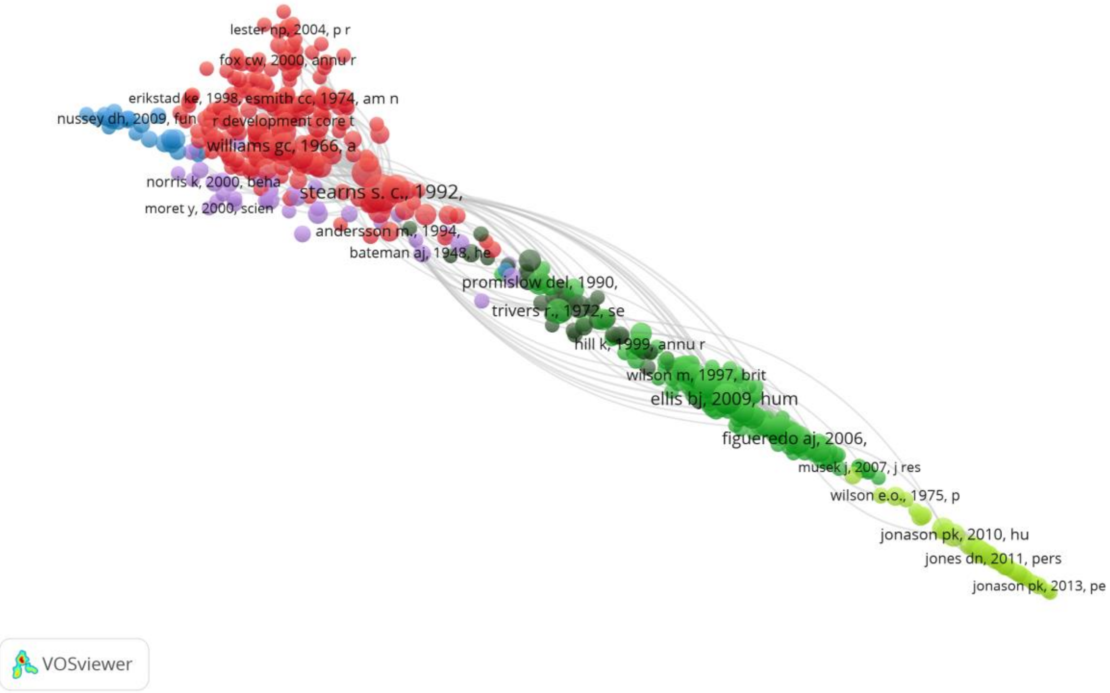

Figure S4. Co-citation maps for the period up to and including 2010 (A) and post-2010 (B). For parameter values see text.

Section 5. Varying the cluster resolution parameter

The cluster detection is controlled by a resolution parameter (with higher resolution representing greater sensitivity to clustering). We chose 0.80 for the main analyses as this gives a reasonable number of interpretable clusters in both time periods. This was a non-pre-registered researcher decision not based on any a priori rationale. We also experimented with other cluster resolution values (table S1).

Table S1. Brief description of cluster structure detected in each time period using different values of the cluster resolution parameter.

Time period	Resolution	Clusters	Notes
Up to and including 2010	0.26	1	Maximal resolution that produces a single cluster
	0.40	2	‘Human’ and ‘non-human’
	0.60	3	‘Birds and mammals’, ‘fish, invertebrates and others’ and ‘human’
	0.80	5	As described in main paper.
	1.00	6	Cluster A4 divided in two. Others unchanged.
Post-2010	0.15	1	Maximal resolution that produces a single cluster
	0.40	2	‘Human’ and ‘non-human’
	0.60	3	‘Birds and mammals’, ‘fish, invertebrates, plants and others’, and ‘human’
	0.80	5	As described in main paper.
	1.00	7	Clusters B1 and B3 each split into two. B2, B4 and B5 unchanged.

The maximum cluster resolution that can be used before distinct clusters were detected was different in the two time periods: 0.26 in the literature up to 2010, but only 0.15 in the more recent period. This, along with the other evidence discussed in the main paper, suggests a greater degree of internal separation in the more recent period. In both time periods, the first cluster division to appear was ‘human’ versus ‘non-human’. At a cluster resolution of 0.60, again in both time periods, the ‘non-human’ cluster split into one focussed on birds and mammals, and another focussed on fish, invertebrates, reptiles, plants, etc. The non-human clusters further divided more finely as the resolution was increased beyond 0.80.

We also examined the maximum cluster resolution that can be used before distinct clusters are detected in the four finer-grained time categories detailed in section 3 of this document. The

resolutions were 0.27 (up to and including 2004); 0.25 (2005-2010); 0.18 (2011-14); and 0.16 (2015-8).

Section 6. Lists of sampled papers by cluster

Cluster A1

McNamara JM, Houston AI (2006) State-dependent life histories. Nature 380:215–221.

Young BE (1996) An experimental analysis of small clutch size in tropical house wrens. Ecology 77:472–488. doi: 10.2307/2265623

Ricklefs RE (2010) Parental Investment and avian reproductive rate: Williams's Principle reconsidered. Am Nat 175:350–361. doi: 10.1086/650371

Murphy MT (2000) Evolution of clutch size in the Eastern Kingbird: Tests of alternative hypotheses. Ecol Monogr 70:1–20. doi: 10.1890/0012-9615(2000)070[0001:EOCSIT]2.0.CO;2

Martin TE, Bassar RDD, Bassar SKK, et al (2006) Life-history and ecological correlates of geographic variation in egg and clutch mass among passerine species. Evolution 60:390–8. doi: 10.1111/j.0014-3820.2006.tb01115.x

Hillstrom L (1995) Body mass reduction during reproduction in the Pied Flycatcher Ficedula hypoleuca: Physiological stress or adaptation for lowered costs of locomotion? Funct Ecol 9:807–817.

Harding AMA, Kitaysky AS, Hall ME, et al (2009) Flexibility in the parental effort of an Arctic-breeding seabird. Funct Ecol 23:348–358. doi: 10.1111/j.1365-2435.2008.01488.x

Hanssen SA, Hasselquist D, Folstad I, Erikstad KE (2005) Cost of reproduction in a long-lived bird: Incubation effort reduces immune function and future reproduction. Proc R Soc B Biol Sci 272:1039–46. doi: 10.1098/rspb.2005.3057

Griebeler EM, Caprano T, Böhning-Gaese K (2010) Evolution of avian clutch size along latitudinal gradients: Do seasonality, nest predation or breeding season length matter? J Evol Biol 23:888–901. doi: 10.1111/j.1420-9101.2010.01958.x

Brommer J, Kokko H, Pietiäinen H (2000) Reproductive effort and reproductive values in periodic environments. Am Nat 155:454–472. doi: 10.1086/303335

Cluster A2

Low BS, Anderson K, Simon C (2003) The biodemography of human reproduction and fertility. In: Rodgers JL, Kohler H-P (eds) *The Biodemography of Human Reproduction and Fertility*. Kluwer Academic, Boston, pp 105–34

Hill EM, Ross LT, Low BS (1997) The role of future unpredictability in human risk-taking. Hum Nat 8:287–325. doi: 10.1007/BF02913037

Low BS, Simon CP, Anderson KG (2002) An evolutionary ecological perspective on demographic transitions: Modeling multiple currencies. Am J Hum Biol 14:149–167. doi: 10.1002/ajhb.10043

Kennedy GE (2003) Palaeolithic grandmothers? Life history theory and early Homo. J R Anthropol Inst 9:549–572. doi: 10.1111/1467-9655.00163

Kaplan HS (1996) A theory of fertility and parental investment in traditional and modern human societies. Yearb Phys Anthropol 39:91–135. doi: 10.1002/(SICI)1096-8644(1996)23+<91::AID-AJPA4>3.0.CO;2-C

Coall DA, Chisholm JS (2010) Reproductive development and parental investment during pregnancy: Moderating influence of mother's early environment. Am J Hum Biol 22:143–153. doi: 10.1002/ajhb.20965

Borgerhoff Mulder M (2000) Optimizing offspring: the quantity–quality tradeoff in agropastoral Kipsigis. Evol Hum Behav 21:391–410. doi: 10.1016/S1090-5138(00)00054-4

Alvarez HP (2000) Grandmother hypothesis and primate life histories. Am J Phys Anthropol 450:435–450.

Chisholm JS (1999) Attachment and time preference: Relations between early stress and sexual behavior in a sample of American university women. Hum Nat 10:51–83. doi: 10.1007/s12110-999-1001-1

Hawkes K (2003) Grandmothers and the evolution of human longevity. Am J Hum Biol 15:380–400. doi: 10.1002/ajhb.10156

Cluster A3

Brommer JE (2000) The evolution of fitness in life-history theory. Biol Rev 75:377–404. doi: 10.1111/j.1469-185X.2000.tb00049.x

Mangel M, Munch SB (2005) A life-History perspective on short- and long-term consequences of compensatory growth. Am Nat 166:E155–E176. doi: 10.1086/444439

Lytle DA (2001) Disturbance regimes and life-history evolution. Am Nat 157:525–536. doi: 10.1086/319930

Evenor D, Levi-Nissim A, Afgin L, et al (1997) Regeneration of plantlets and bulblets from explants and callus of Allium aflatunense cultivars and selection from indigenous Israeli Allium ampeloprasum. Acta Hortic 430:325–330. doi: 10.1007/s12649-017-0038-3

Gervasi SS, Foufopoulos J (2008) Costs of plasticity: Responses to desiccation decrease post-metamorphic immune function in a pond-breeding amphibian. Funct Ecol 22:100–108. doi: 10.1111/j.1365-2435.2007.01340.x

Dunlop ES, Heino M, Dickmann U (2009) Eco-genetic modeling of contemporary life-history evolution. Ecol Appl 19:1815–1834.

Blanckenhorn WU (1998) Adaptive phenotypic plasticity in growth, development, and body size in the Yellow Dung Fly. Evolution (N Y) 52:1394–1407.

Berner D, Blanckenhorn WU (2006) Grasshopper ontogeny in relation to time constraints: Adaptive divergence and stasis. J Anim Ecol 75:130–139. doi: 10.1111/j.1365-2656.2005.01028.x

Arlinghaus R, Matsumura S, Dieckmann U (2009) Quantifying selection differentials caused by recreational fishing: development of modeling framework and application to reproductive investment in pike (Esox lucius). Evol Appl 2:335–355. doi: 10.1111/j.1752-4571.2009.00081.x

Rose KA, Island D, Winemiller KO (2001) Compensatory density-dependence in fish populations: Importance, controversy, understanding, and prognosis. Fish Fish 2:293–327.

Cluster A4

Warne RW, Charnov EL (2008) Reproductive allometry and the size-number trade-off for lizards. Am Nat 172:E80–E98. doi: 10.1086/589880

Wapstra E, Swain R, Journal S, Jun N (2014) Society for the study of Amphibians and Reptiles geographic and annual variation in life-history traits in a temperate zone Australian skink. J Herpetol 35:194–203.

Shine R, Schwarzkopf L (1992) The evolution of reproductive effort in lizards and snakes. Evolution 46:62. doi: 10.2307/2409805

Miller LK, Brooks R (2005) The effects of genotype, age, and social environment on male ornamentation, mating behavior, and attractiveness. Evolution 59:2414–2425. doi: 10.1111/j.0014-3820.2005.tb00951.x

Glazier DS (1999) Trade-offs between reproductive and somatic (storage) investments in animals: A comparative test of the Van Noordwijk and De Jong model. Evol Ecol 13:539–555. doi: 10.1023/A:1006793600600

Gilbert JDJ, Manica A (2010) Parental care trade-offs and life-history relationships in insects. Am Nat 176:212–226. doi: 10.1086/653661

Cox RM, Calsbeek R (2010) Severe costs of reproduction persist in Anolis lizards despite the evolution of a single-egg clutch. Evolution 64:1321–1330. doi: 10.1111/j.1558-5646.2009.00906.x

Charnov EL, Warne R, Moses M (2007) Lifetime reproductive effort. Am Nat 170:E129–E142. doi: 10.1086/522840

Bridges TS (1996) Fitness consequences of maternal effects in Streblospio benedicti (annelida: polychaeta). Am Zool 36:132–146. doi: 10.1093/icb/36.2.132

Angilletta, Jr. MJ, Niewiarowski PH, Dunham AE, et al (2004) Bergmann's clines in ectotherms: Illustrating a life-history perspective with Sceloporine lizards. Am Nat 164:E168–E183. doi: 10.1086/425222

Cluster A5

Sanz-Aguilar A, Tavecchia G, Pradel R, et al (2008) The cost of reproduction and experience dependent vital rates in a small petrel. Ecology 89:3195–3203. doi: 10.1890/08-0431.1

Oli MK (2004) The fast-slow continuum and mammalian life-history patterns: An empirical evaluation. Basic Appl Ecol 5:449–463. doi: 10.1016/j.baae.2004.06.002

Hadley GL, Rotella JJ, Garrott RA (2007) Evaluation of reproductive costs for Weddell seals in Erebus Bay, Antarctica. J Anim Ecol 76:448–458. doi: 10.1111/j.1365-2656.2007.01219.x
Hadley GL, Rotella JJ, Garrott RA, Nichols JD (2006) Variation in probability of first reproduction of Weddell seals. J Anim Ecol 75:1058–70. doi: 10.1111/j.1365-2656.2006.01118.x
Garel M, Solberg EJ, Sæther B, et al (2009) Age, size, and spatiotemporal variation in ovulation patterns of a seasonal breeder, the Norwegian moose (Alces alces). Am Nat 173:89–104. doi: 10.1086/593359
Ferguson SH (2002) Using survivorship curves to estimate age of first reproduction in moose Alces alces. Wildlife Biol 8:129–136. doi: 10.1070/MC2005v015n06ABEH002246
Berube CH, Festa-Bianchet M, Jorgenson JT (1999) Individual differences, longevity, and reproductive senescence in Bighorn ewes. Ecology 80:2555–2565.
Beauplet G, Barbraud C, Dabin W, et al (2006) Age-specific survival and reproductive performances in fur seals: evidence of senescence and individual quality. Oikos 112:430–41. doi: 10.1007/978-3-658-02694-3
Beauplet G, Barbraud C, Chambellant M, Guinet C (2005) Interannual variation in the post-weaning and juvenile survival of subantarctic fur seals: Influence of pup sex, growth rate and oceanographic conditions. J Anim Ecol 74:1160–1172. doi: 10.1111/j.1365-2656.2005.01016.x
Dobson F S, Oli, M K (2007). Fast and slow life histories of mammals. Ecoscience 14: 292-297. doi: 10.2980/1195-6860(2007)14[292:FASLHO]2.0.CO;2

Cluster B1

Sofaer HR, Sillett TS, Yoon J, et al (2018) Offspring growth and mobility in response to variation in parental care: a comparison between populations. J Avian Biol 49:1–14. doi: 10.1111/jav.01646
Balbontín J, Møller AP (2015) Environmental conditions during early life accelerate the rate of senescence in a short-lived passerine bird. Ecology 96:948–959. doi: 10.1890/14-1274.1
Blount JD, Vitikainen EIK, Stott I, Cant MA (2016) Oxidative shielding and the cost of reproduction. Biol Rev 91:483–497. doi: 10.1111/brv.12179
Chambert T, Rotella JJ, Higgs MD, Garrott RA (2013) Individual heterogeneity in reproductive rates and cost of reproduction in a long-lived vertebrate. Ecol Evol 3:2047–2060. doi: 10.1002/ece3.615
Fowler MA, Williams TD (2017) A Physiological Signature of the Cost of Reproduction Associated with Parental Care. Am Nat 190:000–000. doi: 10.1086/694123
Hua F, Sieving KE, Fletcher RJ, Wright CA (2014) Increased perception of predation risk to adults and offspring alters avian reproductive strategy and performance. Behav Ecol 25:509–519. doi: 10.1093/beheco/aru017
Neggazi SA, Noreikiene K, Öst M, Jaatinen K (2016) Reproductive investment is connected to innate immunity in a long-lived animal. Oecologia 182:347–356. doi: 10.1007/s00442-016-3657-7
Paterson JT, Rotella JJ, Mannas JM, Garrott RA (2016) Patterns of age-related change in reproductive effort differ in the pre-natal and post-natal periods in a long-lived mammal. J Anim Ecol 85:1540–1551. doi: 10.1111/1365-2656.12577
Paterson JT, Rotella JJ, Link WA, Garrott R (2018) Variation in the vital rates of an Antarctic marine predator: the role of individual heterogeneity. Ecology 99:2385–2396. doi: 10.1002/ecy.2481
Vedder O, Bouwhuis S (2018) Heterogeneity in individual quality in birds: overall patterns and insights from a study on common terns. Oikos 127:719–727. doi: 10.1111/oik.04273

Cluster B2

Griskevicius V, Delton AW, Robertson TE, Tybur JM (2011) Environmental contingency in life history strategies: the influence of mortality and socioeconomic status on reproductive timing. J Pers Soc Psychol 100:241–254. doi: 10.1037/a0021082
Mittal C, Griskevicius V (2014) Sense of Control Under Uncertainty Depends on People's Childhood Environment: A Life History Theory Approach. J Pers Soc Psychol 107:621–637. doi: 10.1037/a0037398
Webster GD, Graber JA, Gesselman AN, et al (2014) Life history theory of father absence and menarche: A meta-analysis. Evol Psychol 12:273–294. doi: 10.1177/147470491401200202
Richardson GB, Chen CC, Dai CL, et al (2014) Life history strategy and young adult substance use. Evol Psychol 12:932–957. doi: 10.1177/147470491401200506

Gettler LT, McDade TW, Bragg JM, et al (2015) Developmental energetics, sibling death, and parental instability as predictors of maturational tempo and life history scheduling in males from Cebu, Philippines. Am J Phys Anthropol 158:175–184. doi: 10.1002/ajpa.22783
Griskevicius V, Delton AW, Robertson TE, Tybur JM (2011) Environmental contingency in life history strategies: the influence of mortality and socioeconomic status on reproductive timing. J Pers Soc Psychol 100:241–254. doi: 10.1037/a0021082
Kubinski JS, Chopik WJ, Grimm KJ (2017) Change across the lifespan in a psychological measure of life history strategy. Evol Hum Behav 38:434–441. doi: 10.1016/j.evolhumbehav.2017.04.005
Neel R, Kenrick DT, White AE, Neuberg SL (2016) Individual differences in fundamental social motives. J Pers Soc Psychol 110:887–907. doi: 10.1037/pspp0000068
Richardson GB, Sanning BK, Lai MHC, et al (2017) On the psychometric study of human life history strategies: State of the science and evidence of two independent dimensions. Evol Psychol 15:1–24. doi: 10.1177/1474704916666840
Xu Y, Norton S, Rahman Q (2018) Early life conditions, reproductive and sexuality-related life history outcomes among human males: A systematic review and meta-analysis. Evol Hum Behav 39:40–51. doi: 10.1016/j.evolhumbehav.2017.08.005

Cluster B3

Pettersen AK, White CR, Bryson-Richardson RJ, Marshall DJ (2018) Does the cost of development scale allometrically with offspring size? Funct Ecol 32:762–772. doi: 10.1111/1365-2435.13015
Marty L, Rochet MJ, Ernande B (2014) Temporal trends in age and size at maturation of four North Sea gadid species: Cod, haddock, whiting and Norway pout. Mar Ecol Prog Ser 497:179–197. doi: 10.3354/meps10580
Marty L, Dieckmann U, Rochet M-J, Ernande B (2011) Impact of environmental covariation in growth and mortality on evolving maturation reaction norms. Am Nat 177:E98–E118. doi: 10.1086/658988
Hughes PW, Simons AM (2014) The continuum between semelparity and iteroparity: Plastic expression of parity in response to season length manipulation in Lobelia inflata. BMC Evol Biol 14:1–11. doi: 10.1186/1471-2148-14-90
Hughes PW, Simons AM (2014) Changing reproductive effort within a semelparous reproductive episode. Am J Bot 101:1323–1331. doi: 10.3732/ajb.1400283
Hou C, Bolt K, Bergman A (2011) A general life history theory for effects of caloric restriction on health maintenance. BMC Syst Biol. doi: 10.1186/1752-0509-5-78
Honsey AE, Staples DF, Venturelli PA (2017) Accurate estimates of age at maturity from the growth trajectories of fishes and other ectotherms: Ecol Appl 27:182–192. doi: 10.1002/eap.1421
Furness AI, Reznick DN (2014) The comparative ecology of a killifish (Rivulus hartii) across aquatic communities differing in predation intensity. Evol Ecol Res 16:249–265.
Filin I (2015) The relation between maternal phenotype and offspring size, explained by overhead material costs of reproduction. J Theor Biol 364:168–178. doi: 10.1016/j.jtbi.2014.09.007
Cameron H, Monro K, Marshall DJ (2017) Should mothers provision their offspring equally? A manipulative field test. Ecol Lett 20:1025–1033. doi: 10.1111/ele.12800

Cluster B4

Volk AA, Atkinson JA (2013) Infant and child death in the human environment of evolutionary adaptation. Evol Hum Behav 34:182–192. doi: 10.1016/j.evolhumbehav.2012.11.007
Urlacher SS, Ellison PT, Sugiyama LS, et al (2018) Tradeoffs between immune function and childhood growth among Amazonian forager-horticulturalists. Proc Natl Acad Sci 115:201717522. doi: 10.1073/pnas.1717522115
Thompson ME (2016) Faster reproductive rates trade off against offspring growth in wild chimpanzees. Proc Natl Acad Sci U S A 113:7780–5. doi: 10.1073/pnas.1522168113
Skjærvø GR, Røskaft E (2013) Menopause: No support for an evolutionary explanation among historical Norwegians. Exp Gerontol 48:408–413. doi: 10.1016/j.exger.2013.02.001
Schniter E, Gurven M, Kaplan HS, et al (2015) Skill ontogeny among Tsimane forager-horticulturalists. Am J Phys Anthropol 158:3–18. doi: 10.1002/ajpa.22757

Mattison SM, Neill DB (2013) The effects of residential ecology on patterns of child work and mother's reproductive success among indo-fijians. Evol Hum Behav 34:207–215. doi: 10.1016/j.evolhumbehav.2013.01.002

Macho GA, Lee-Thorp JA (2014) Niche partitioning in sympatric Gorilla and Pan from Cameroon: Implications for life history strategies and for reconstructing the evolution of hominin life history. PLoS One. doi: 10.1371/journal.pone.0102794

Kramer KL, Veile A, Otárola-Castillo E (2016) Sibling competition & growth tradeoffs. Biological vs. statistical significance. PLoS One 11:1–17. doi: 10.1371/journal.pone.0150126

Jones JH, Bird RB (2014) The marginal valuation of fertility. Evol Hum Behav 35:65–71. doi: 10.1016/j.evolhumbehav.2013.10.002

Bürkli A, Postma E (2014) Genetic constraints underlying human reproductive timing in a premodern swiss village. Evolution (N Y) 68:526–537. doi: 10.1111/evo.12287

Cluster B5

Jones DN, De Roos MS (2017) Machiavellian flexibility in negative mate retention. Pers Relatsh 24:265–279. doi: 10.1111/pere.12181

Jonason PK, Icho A, Ireland K (2016) Resources, harshness, and unpredictability: The socioeconomic conditions associated with the dark triad traits. Evol Psychol 14:1–11. doi: 10.1177/1474704915623699

Jonason PK, Ferrell JD (2016) Looking under the hood: The psychogenic motivational foundations of the Dark Triad. Pers Individ Dif 94:324–331. doi: 10.1016/j.paid.2016.01.039

Jonason PK, Baughman HM, Carter GL, Parker P (2015) Dorian Gray without his portrait: Psychological, social, and physical health costs associated with the Dark Triad. Pers Individ Dif 78:5–13. doi: 10.1016/j.paid.2015.01.008

Jonason PK, Richardson EN, Potter L (2015) Self-reported creative ability and the Dark Triad traits: An exploratory study. Psychol Aesthetics, Creat Arts 9:488–494. doi: 10.1037/aca0000037

Jonason PK, Li NP, Czarna AZ (2013) Quick and dirty: Some psychosocial costs associated with the dark triad in three countries. Evol Psychol 11:172–185. doi: 10.1177/147470491301100116

Jonason PK, Schmitt DP (2012) What have you done for me lately? Friendship-selection in the shadow of the dark triad traits. Evol Psychol 10:400–421. doi: 10.1177/147470491201000303

Czarna AZ, Jonason PK, Dufner M, Kossowska M (2016) The Dirty Dozen scale: Validation of a polish version and extension of the nomological net. Front Psychol 7:1–12. doi: 10.3389/fpsyg.2016.00445

Book A, Visser BA, Volk AA (2015) Unpacking "evil": Claiming the core of the Dark Triad. Pers Individ Dif 73:29–38. doi: 10.1016/j.paid.2014.09.016

Baughman HM, Jonason PK, Veselka L, Vernon PA (2014) Four shades of sexual fantasies linked to the Dark Triad. Pers Individ Dif 67:47–51. doi: 10.1016/j.paid.2014.01.034

Section 7. Information on the clusters (tables S1 and S2)

Table S1. Data on the clusters identified by the bibliographic coupling analysis, period up to and including 2010.

Cluster	n	Name	Topics	Taxa	Formal model in sample papers belonging to cluster	Fast-slow axis	References most cited by papers belonging to cluster	Formal model in ten sources most cited by papers belonging to cluster
A1	120	Birds	Reproductive effort, ageing	Birds 8/8	5 / 10	0 / 10	1. *ELH-S*; 2. *ELH-R*; 3. Drent, R. H. (1980) *Ardea* 68: 225; 4. Clutton-Brock, T. C. (1991). *Evolution of Parental Care* (Princeton); 5. Deerenberg, C. (1997). *Proc. Roy. Soc. B* 264: 1021; 6. Linden, M. (1989). *Trends. Ecol. Evol.* 4: 367; 7. Martin, T.E. (1987). *Ann. Rev. Ecol. Systematics* 18: 453; 8. Lack, D. (1968). *Ecological Adaptations for Breeding in Birds* (Chapman Hall; 9. Lessells, C. M. (1991) in: *Behavioural Ecology: An Evolutionary Approach* (Blackwell); 10. Sheldon, B. C. (1996). *Trends. Ecol. Evol.* 11: 317	3 / 10
A2	115	Humans	Reproductive effort, ageing, childhood psychosocial experience	Humans 10 / 10<br>Non-human primates 1/10	3 / 10	1/10	1. *ELH-S*; 2. Belsky, J. (1991). *Child Dev.* 62: 647; 3. Charnov, E. L. (1993). *Life History Invariants* (Oxford); 4. Chisholm, J.S. (1993). *Curr. Anthro.* 34: 1; 5. Kaplan, H.S. (2000). *Evol. Anthro.* 9: 156; 6. Hill, K. (1996). *Ache Life History* (Routledge); 7. Hawkes, K. (1998). *PNAS* 95: 1336; 8. Hill, K. (1999). *Ann. Rev. Anthro.* 28: 397; 9. *ELH-R*; 10. Bogin, B. (1999). *Patterns of Human Growth* (Cambridge).	4 / 10
A3	109	Invertebrates and others	Growth, maturation, theory	Invertebrates 3 / 6<br>Fish 2 / 6<br>Amphibians 1 / 6	6 / 10	0 / 10	1. *ELH-R*; 2. *ELH-S*; 3. Abrams, P.A. (1996). *Am. Nat.* 147: 381; 4. Charlesworth, B. (1980). *Evolution in Age-Structured Populations* (Cambridge); 5. Arendt, J.D. (1997). *Q. Rev. Biol.* 72: 149; 6. Cole, L.C. (1954). *Q. Rev. Biol.* 29: 103; 7. Stearns, S.C. (1992). *Evolution* 40: 893; 8. Fisher, R.A. (1930). *The Genetical Theory of Natural Selection* (Oxford); 9. Stearns, S.C. (1976). *Q. Rev. Biol.* 51: 3; 10. Atkinson, D. (1994). *Adv. Ecol. Res.* 25: 1.	8 / 10
A4	93	Reptiles and others	Reproductive effort, parental investment	Reptiles 7 /10<br>Fish 2 / 10<br>Invertebrates 2 / 10<br>Non-primate mammals 1 / 10<br>Non-human primates 1 / 10	2 / 10	0 / 10	1. *ELH-R*; 2. *ELH-S*; 3. Reznick, D. (1985). *Oikos* 44: 257; 4. Smith, C.C. (1974). *Am. Nat.* 108: 499; 5. *LHE*; 6. Gadgil, M. (1970). *Am. Nat.* 104: 1; 7. Williams, G.C. (1966). *Am. Nat.* 100: 687; 8. Houle, D. (1992). *Genetics* 130: 195; 9. McGinley, M.A. (1987). *Am. Nat.* 130: 370; 10. Parker, G.A. (1986). *Am. Nat.* 128: 573.	9 / 10
A5	63	Mammals	Reproductive effort, ageing, maturation, fast-slow continuum	Non-primate mammals 9 / 10<br>Non-human primates 2 / 10<br>Birds 1 / 10	0 / 10	3 / 10	1. Burnham, K. P. (1998). *Model Selection and Multimodel Inference* (Springer); 2. LeBreton. J.D. (1992). *Ecol. Monographs* 62: 67; 3. *ELH-S*; 4. Brownie, C. (1993). *Biometrics* 49: 1173; 5. Caswell, H. (2001). *Matrix Population Models* (Oxford); 6. Cole, L.C. (1954). *Q. Rev. Biol.* 29: 103; 7. Clutton-Brock, T. C. (1982). *Red Deer* (Chicago); 8. Gaillard, J.M. *Ann. Rev. Ecol. Systematics* 31: 367; 9. Cam, E. (1998) *Ecology* 79: 2917; 10. Cam, E. (2002). *Am. Nat.* 159: 96.	3 / 10

Notes: Top cited references are in descending order of citations. First author name only is shown. *ELH-S*: Stearns, S.C. (1992). *The Evolution of Life Histories* (Oxford); *ELH-R*: Roff, D. (1992). *The Evolution of Life Histories* (Chapman and Hall); *LHE*: Roff, D. (2002). *Life History Evolution* (New York: Oxford University Press). Note that taxa numbers do not necessarily add to 10 as some papers contain only models, and others contain data from multiple taxa.

Table 2. Data on the clusters identified by the bibliographic coupling analysis, post 2010.

Cluster	N	Name	Topics	Taxa	Formal model in sample papers belonging to cluster	Fast-slow axis	References most cited by papers belonging to cluster	Formal model in ten sources most cited by papers belonging to cluster
B1	184	Birds and mammals	Reproductive effort, ageing, parental investment	Birds 7/10<br>Non-primate mammals 4/10	0 / 10	2/10	1. *ELH-S*; 2. Burnham, K. P. (1998). *Model Selection and Multimodel Inference* (Springer); 3. Harshman, L.G. (2007). *Trends. Ecol. Evol.* 22: 80; 4. Alonso-Alvarez, C. (2004). *Ecol. Letters* 7: 363; 5. Clutton-Brock, T.H. (1984). *Am. Nat.* 123:212; 6. Bergeron, P. (2011). *Funct. Ecol.* 25: 1063; 7. Clutton-Brock, T. C. (1991). *Evolution of Parental Care* (Princeton); 8. Garratt, M. (2011). *Proc. Roy. Soc. B* 278: 1098; 9. Van Noordwijk, A.J. (1986). *Am. Nat.* 128: 137; 10. Williams, G.C. (1966). *Am. Nat.* 100: 687.	3 / 10
B2	155	Human developmental/ personality	Childhood psychosocial experience in relation to maturation and psychological traits	Humans 10/10	0/10	10/10	1. Ellis, B.J. (2009). *Hum. Nat.* 20: 204; 2. Belsky, J. (1991). *Child Dev.* 62: 647; 3. Figueredo, A.J. (2006). *Dev. Rev.* 26: 243; 4. Chisholm, J.S. (1993). *Curr. Anthro.* 34: 1; 5. Ellis, B.J. (2004). *Psych. Bull.* 130: 920; 6. Belsky, J. (2012). *Dev. Psych.* 48: 662; 7. Figueredo, A.J. (2004). *Soc. Biol.* 51: 121; 8. Del Giudice, M. (2009). *Behav. Brain. Sci* 32: 1; 9. Brumbach, B.H. (2009). *Hum. Nat.* 20: 25; 10. Figeredo, A.J. (2005). *Pers. Ind. Diff.* 39: 1349.	0 / 10
B3	90	Fish and others	Reproductive effort, growth, ageing, parental investment, energetics	Fish 4/7<br>Plants 2/7<br>Invertebrates 1/7	3/10	0/10	1. *ELH-S*; 2. *ELH-R*; 3. *LHE*; 4. Bell, G. (1980). *Am. Nat.* 116: 45; 5. Bernado, J. (1996). *Am. Zool.* 36: 216; 6. Smith, C. C. (1974). *Am. Nat.* 108: 499; 7. Allen, R.M. (2008). *Am. Nat.* 171: 225; 8. Berkeley, S.A. (2004). *Ecology* 85: 1258; 9. Caswell, H. (2001). *Matrix Population Models* (Oxford); 10. Conover, D. H. (2002). *Science* 297: 94.	5 / 10
B4	40	Human evolution	Reproductive effort, growth, mortality, immunity, alloparenting, skill acquisition	Humans 8/10<br>Non-human primates 3/10	0/10	2/10	1. Kaplan, H.S. (2000). *Evol. Anthro.* 9: 156; 2. Hawkes, K. (1998). *PNAS* 95: 1336; 3. Hill, K. (1996). *Ache Life History* (Routledge); 4. *ELH-S*; 5. Bogin, B. (1997). *Yearb. Phys. Anthro.* 40: 63; 6. Charnov, E.L. (1993). *Evol. Anthro.* 1: 191; 7. Gurven, M. (2006). *Proc. Roy. Soc. B* 273: 835; 8. Hamilton, W.D. (1966). *J. Theor. Bio.* 12: 12; 9. Kaplan, H.S. (2002). *PNAS* 99: 10221; 10. Kuzawa, C.W. (2012). *Curr. Anthro.* 53: 369.	4 / 10
B5	29	Dark triad personality	'Dark triad' personality traits (Machiavellianism, narcissism, and psychopathy)	Humans 10/10	0/10	10/10	1. Jonason, P.K. (2009). *Eur. J. Pers.* 23: 5; 2. Jonason, P.K. (2010). *Hum. Nat.* 21: 428; 3. Paulhus, D.L. (2002). *J. Res. Pers.* 36: 556; 4. Jonason, P.K. (2010). *Psychol. Assessment* 22: 420; 5. Christie, R. (1970). *Studies in Machiavellianism* (Academic Press); 6. Figueredo, A.J. (2006). *Dev. Rev.* 26: 243; 7. Jonason, P.K. (2012). *Rev. Gen. Psych.*16: 192; 8. Jonason, P.K. (2010). *Pers. Ind. Diff.* 49: 611; 9. Jonason, P.K. (2012). *Pers. Ind. Diff.* 52: 521; 10. Jones, D.N. (2011). *Pers. Ind. Diff.* 51: 679.	0 / 10

Notes: Top cited references are in descending order of citations. First author name only is shown. *ELH-S*: Stearns, S.C. (1992). *The Evolution of Life Histories* (Oxford); *ELH-R*: Roff, D. (1992). *The Evolution of Life Histories* (Chapman and Hall); *LHE*: Roff, D. (2002). *Life History Evolution* (New York: Oxford University Press). Note that taxa numbers do not necessarily add to 10 as some papers contain only models, and others contain data from multiple taxa. Note that taxa numbers do not necessarily add to 10 as some papers contain only models, and others contain data from multiple taxa.

References cited (this document)

1. Stearns, S. 1976 Life history tactics: A review of the ideas. *Q. Rev. Biol.* **51**, 3–47.

2. Smith, C. C. & Fretwell, S. D. 1974 The optimal balance between size and number of offspring. *Am. Nat.* **108**, 499–506. (doi:10.1086/282929)

3. Stearns, S. C. 1980 A new view of life-history evolution. *Oikos* **35**, 266–281.

4. Charnov, E. L. 1979 Natural selection and sex change in pandalid shrimp : Test of a life-history theory. *Am. Nat.* **113**, 715–734.

5. Skirnisson, K. 2016 Association of helminth infections and food consumption in common eiders Somateria mollissima in Iceland. *J. Sea Res.* **113**, 132–141. (doi:10.1016/j.seares.2016.06.002)

6. Zhou, K., Sun, S., Wang, M., Wang, S. & Li, C. 2016 Differences in the physiological processes of *Calanus sinicus* inside and outside the Yellow Sea Cold Water Mass. *J. Plankton Res.* **38**, 551–563. (doi:10.1093/plankt/fbw011)

7. Helle, S., Lummaa, V. & Jokela, J. 2004 Accelerated immunosenescence in preindustrial twin mothers. *Proc. Natl. Acad. Sci. USA* **101**, 12391–12394. (doi:10.1073/pnas.0402215101)

8. Chisholm, J. S. 1996 The evolutionary ecology of attachment organization. *Hu Nat* **7**, 1–37. (doi:10.1007/BF02733488)

9. Rushton, J. P. 1985 Differential K theory: The sociobiology of individual and group differences. *Pers. Individ. Dif.* **6**, 441–452. (doi:10.1016/0191-8869(85)90137-0)

10. Boyack, K. W. & Klavans, R. 2010 Co-citation analysis, bibliographic coupling, and direct citation: Which citation approach represents the research front most accurately? *J. Am. Soc. Inf. Sci. Technol.* **61**, 2389–404. (doi:10.1002/asi)
